# Population Genetic Analyses of *Helicobacter pylori* Isolates from Gambian Adults and Children

**DOI:** 10.1371/journal.pone.0109466

**Published:** 2014-10-13

**Authors:** Ousman Secka, Yoshan Moodley, Martin Antonio, Douglas E. Berg, Mary Tapgun, Robert Walton, Archibald Worwui, Vivat Thomas, Tumani Corrah, Julian E. Thomas, Richard A. Adegbola

**Affiliations:** 1 Vaccinology, Medical Research Council Unit, Fajara, The Gambia; 2 Department of Integrative Biology and Evolution, Konrad Lorenz Institute for Ethology, University of Veterinary Medicine Vienna, Vienna, Austria; 3 Department of Molecular Microbiology, Washington University School of Medicine, St. Louis, Missouri, United States of America; 4 Department of Medicine, University of California San Diego, La Jolla, California, United States of America; 5 Clinical Services Department, Medical Research Council Unit, Fajara, The Gambia; 6 Department of Paediatric Gastroenterology Great North Children's Hospital, Royal Victoria Infirmary, Newcastle upon Tyne, Tyne and Wear, United Kingdom; 7 Bacterial Diseases Programme, Medical Research Council Unit, Fajara, The Gambia; Institut Pasteur Paris, France

## Abstract

The gastric pathogen *Helicobacter pylori* is one of the most genetically diverse of bacterial species. Much of its diversity stems from frequent mutation and recombination, preferential transmission within families and local communities, and selection during persistent gastric mucosal infection. MLST of seven housekeeping genes had identified multiple distinct *H. pylori* populations, including three from Africa: hpNEAfrica, hpAfrica1 and hpAfrica2, which consists of three subpopulations (hspWAfrica, hspCAfrica and hspSAfrica). Most detailed *H. pylori* population analyses have used strains from non-African countries, despite Africa's high importance in the emergence and evolution of humans and their pathogens. Our concatenated sequences from seven *H. pylori* housekeeping genes from 44 Gambian patients (MLST) identified 42 distinct sequence types (or haplotypes), and no clustering with age or disease. STRUCTURE analysis of the sequence data indicated that Gambian *H. pylori* strains belong to the hspWAfrica subpopulation of hpAfrica1, in accord with Gambia's West African location. Despite Gambia's history of invasion and colonisation by Europeans and North Africans during the last millennium, no traces of Ancestral Europe1 (AE1) population carried by those people were found. Instead, admixture of 17% from Ancestral Europe2 (AE2) was detected in Gambian strains; this population predominates in Nilo-Saharan speakers of North-East Africa, and might have been derived from admixture of hpNEAfrica strains these people carried when they migrated across the Sahara during the Holocene humid period 6,000–9,000 years ago. Alternatively, shared AE2 ancestry might have resulted from shared ancestral polymorphisms already present in the common ancestor of sister populations hpAfrica1 and hpNEAfrica.

## Introduction

*H. pylori* is a genetically diverse Gram negative micro-aerophilic bacterial species that chronically infects some half of all humans worldwide [1]. It is implicated in chronic gastritis, gastroduodenal ulcers and gastric cancer [2], [3] and also increases the risk of infection by diarrheal pathogens [4], which may lead to infant malnutrition with growth faltering [5] in low income societies. Despite this, most infections are benign, and some may even be beneficial [6], [7]. The risk of infection resulting in overt disease is likely to be determined by *H. pylori* genotype in combination with other variables such as human genotype and physiology, nutrition and environmental factors.

*H. pylori* is usually acquired in childhood [8] and can persist for life unless eradicated by antibiotics [9]. A prevalence of ≥80% is typical in developing nations [10]–[13], but has fallen dramatically in industrialized countries during the last century (currently, in the range of 20%), probably due to major improvements in hygiene and sanitation [14]. Transmission is predominantly intrafamilial with a low risk of adult infection in industrialized countries [15], [16], whereas transmission within the local community is frequent in developing countries, with new infections often occurring in adults as well as in children [17].

Independent *H. pylori* isolates typically differ by some 2% or more in DNA sequence, which allows different strains to be distinguished readily by the arbitrarily primed PCR (RAPD) method, wherein each strain yields a characteristic pattern of DNA fragments different from those of nearly all other independent strains [18], or by the sequencing of one or more housekeeping genes. As an extension of this approach, multilocus sequence typing (MLST; typically sequencing seven such genes) soon provided early indications that different sets of *H. pylori* genotypes predominated in different human populations [19], [20]. Although high sequence variability confounded the use of MLST in *H. pylori*, more sophisticated nucleotide analyses of DNA sequences have since provided more refined demonstrations both of major geographic or human population differences among *H. pylori* populations and also admixture often linked to human migrations [21], [22]. This great diversity within and between populations can be ascribed to *H. pylori*'s high rates of mutation and recombination [23], coupled with its having chronically infected humans for many thousands of years, its transmission being predominantly within families or local communities, and the tendency of any person's gastric mucosal infection to persist for decades if not treated with antimicrobials. This epidemiologic pattern resulted in the isolation of populations from each other by distance, and allowed mutational divergence by random genetic drift, and selection for locally adapted genotypes. The sequences of housekeeping genes of strains from many parts of the world identified three African *H. pylori* populations, which may be of particular importance to the Gambian strain analyses presented here, designated hpAfrica1, hpAfrica2 and hpNEAfrica [21], [22], [24]. Four further populations, hpEurope, hpEAsia, hpAsia2 and hpSahul, were detected in the rest of the world. More focussed analyses distinguished three hpAfrica1 subpopulations, designated hspWAfrica (Western and North-Western Africa), hspCAfrica (Cameroon) and hspSAfrica (South Africa), and two hpNEAfrica subpopulations - hspEastNEAfrica and hspCentralNEAfrica [25]. The distribution of the various hpAfrica1 subpopulations may reflect the expansion of the Bantu people throughout subSaharan Africa from an ancestral homeland in or near present day Nigeria/Cameroon during the last 4000 years. In contrast the hspEastNEAfrica and hspCentralNEAfrica subpopulations found in Algeria and northern Nigeria are thought to reflect migrations of Nilo-Saharan people through what were then central Saharan wetlands during the Holocene humid period some 6,000–9,000 years ago [25].

The Gambia is a small country, some 47 Km wide and 338 Km long, embracing the west-flowing Gambia River in the most western part of Africa, bordered by Senegal to the North, South and East and the Atlantic Ocean to the West. Gambian contacts with Europeans were mostly with the Portuguese and French beginning in the 15^th^ century, and then with the British in the 17^th^ century and lasting until independence from British colonial rule in 1965. The great majority of present-day Gambians are indigenous West Africans, predominantly of the Mandinka, Wollof and Fulani linguistic groups that are also abundant in nearby countries of Senegal, Guinea Bissau, Guinea and Mali. Most Gambians are Muslim, reflecting conversion of residents by Arab traders from North Africa who crossed the Sahara beginning in the 8^th^ century. Given the tendency of *H. pylori* populations to reflect human host migrations, and since the Gambia was also a major source of slaves taken to the Americas and Europe for several centuries until the slave trade ended in the 1800s, it is possible that Gambian strains contributed significantly to *H. pylori*'*s* gene pool in The Americas and Europe. Indeed, a suggestion of West African admixture in European *H. pylori* had emerged in our earlier study of a novel regulatory gene-linked insertion-deletion polymorphism (indel) in Spanish vs. Gambian *H. pylori* strains [26].

It is with this background that we sequenced the MLST genes of *H. pylori* strains from ethnic West African adults and children in The Gambia. These strains are expected to be broadly representative of *H. pylori* of much of West Africa, a relatively unstudied population. Furthermore, given the historical contact with Europeans and North Africans, we may also detect traces of the ancestral European nucleotides carried by these people within our Gambian sample.

## Materials and Methods

### Ethics statement

Ethical approval for this study was provided by The Gambia Government/MRC joint ethics committee and the NIH Division of Microbiology Infectious Diseases (DMID) International Review Board, USA (NIH number DMID 06-0053; MRC Unit, The Gambia IRB registration number: IRB00003943 and Federal Wide Assurance number: FWA 00006873).

### Patients

Gastric biopsies were collected from patients referred for endoscopy at the Medical Research Council Unit (MRC), The Gambia, for routine clinical investigations of symptoms attributable to gastroduodenal disease. All patients who agreed to join this study provided written informed consent; in addition, for children aged less than 18 years, biopsies were obtained after written informed parental consent. The patients belonged to the following ethnic groups: Mandinka (19), Wollof (9), Jola (6), Fulani (5), Sarahule (4), Serere (1).

### Biopsies

Endoscopes were cleaned and sterilized with Cidex (Johnson and Johnson Co) after each use according to standard care at the MRC Unit, The Gambia (SOP-CLS-001). Biopsies from the gastric antrum and corpus of study participants were immediately placed in 1 ml Brain-Heart Infusion (BHI) broth containing 20% glycerol and transported in ice to the laboratory for culture or stored at −70°C until used.

### Bacterial culture

Biopsies were spread on the surface of selective Columbia-Blood agar, incubated at 37°C in a microaerobic atmosphere and processed as previously described [27]. Single *H. pylori* colonies were isolated and confluent growth derived by spreading cells from them was harvested and preserved in BHI broth containing 20% glycerol and stored at −70°C until analysed.

### Sample choice for MLST

Samples were chosen according to successful subculture of individual *H. pylori* colonies. One or more single colonies were isolated from each of 44 patients and used for these analyses. The 44 patients (23 male and 21 female) ranged in age from 18 months to 72 years (mean 32 years) and had the following clinical manifestations: normal endoscopic appearance (29), gastric erosions (6), gastric ulcer (3), and oesophageal ulcer (1). Five of these patients were malnourished children with enteropathy (ages 18–31 months, mean 19 months). Thirty three patients (75%) were from the Greater Banjul Area (GBA) and 11 (25%) were from rural villages. To investigate genetic heterogeneity within the same stomach, multiple single colonies from each of two patients with normal gastroduodenal appearance by endoscopy were tested by MLST: seven colonies (4 antrum and 3 corpus) from a 14 year old subject; and 11 single colonies (6 antrum and 5 corpus) from a 72 year old subject.

### Genomic DNA extraction

Genomic DNA was prepared from confluent *H. pylori* growth using a commercial kit (Qiagen DNA Mini kit, UK) [27].

### PCR to detect *cagA*

PCR was performed to detect the *cagA* virulence gene using previously described primers and methods [27], and PCR products were detected by electrophoresis in a 1.5% agarose gel with ethidium bromide, and band visualization using Gel Doc 2000 (Bio-Rad laboratories, Milan, Italy).

### Housekeeping gene sequencing and MLST

The seven standard MLST genes (*atpA*, *efp*, *mutY*, *ppa*, *trpC*, *ureI* and *yphC*) were amplified and sequenced as detailed in http://pubmlst.org/Helicobacter. The PCR products were sequenced using an ABI Prism 3130X DNA sequencer (Applied Biosystems, USA). Consensus sequences were generated and assembled using DNAstar programme (Lasergene, USA, Version 7). The sequences obtained were submitted to the *H. pylori* MLST database (http://pubmlst.org/Helicobacter) for allele and sequence type identification. Concatenated sequences were aligned and imported into MEGA version 5. Evolutionary history was inferred using the neighbor-joining tree reconstruction method [28]. The percentage of replicate trees in which the associated taxa clustered together in a bootstrap test of 2000 replicates is depicted beside each branch.

### Nucleotide analyses

Calculation of the ratio of non-synonymous to synonymous changes (d_N_/d_S_) was done with the START2 (Sequence Type Analysis and Recombinational Tests Version 2) tool, which uses the method of Nei and Gojobori to estimate parameters [29].

### Analyses of population structure

To determine the relatedness of Gambian *H. pylori* to previously studied strains from elsewhere, 246 strains representing *H. pylori*'s global diversity were selected from the public MLST data base (Table 1). This data set included all strains previously published from West Africa [22], [25], [30]. Reconstruction of a global phylogeny was carried out using neighbor-joining as described above. In addition, two Bayesian population cluster analyses were performed using STRUCTURE V2.3.4 software [21]. First, the no-admixture model was used to determine the overall structure of the Gambian sequence data with respect to predefined modern populations. Then, the linkage model was used to ascertain ancestral components within our Gambian data set. In the unlikely event that Gambian *H. pylori* represent a new population, we set our values for *K* at greater than the known number of modern and ancestral *H. pylori* populations. We carried out three independent runs at 2≤K≤10 for the no-admixture model and three independent runs at 2≤K≤8 for the linkage model. Each run comprised 50,000 iterations, the first half of which were discarded as burnin.

**Table 1:** *H. pylori* strains and populations selected for comparison with 46 Gambian strains.

Country	hpAfrica1	hpNEAfrica	hpEurope	hpEAsia	hpAfrica2	hpSahul	hpAsia2
Senegal	73	0	0	0	0	0	0
Burkina Faso	12	0	0	0	0	0	0
Cameroon	5	0	0	0	0	0	0
Morocco	5	0	2	0	0	0	0
Algeria	1	3	2	0	0	0	0
South Africa	8	0	0	0	16	0	0
Nigeria	0	8	0	0	0	0	0
Ethiopia	0	7	0	0	0	0	0
Somalia	0	2	0	0	0	0	0
Egypt	0	0	3	0	0	0	0
Spain	0	0	33	0	0	0	0
Finland	0	0	9	0	0	0	0
Estonia	0	0	11	0	0	0	0
India	0	0	0	0	0	0	2
Bangladesh	0	0	0	0	0	0	1
Malaysia	0	0	0	0	0	0	7
Papua New Guinea	0	0	0	0	0	6	0
Australia	0	0	0	0	0	6	0
Japan	0	0	0	24	0	0	0
**Totals**	**104**	**20**	**60**	**24**	**16**	**12**	**10**

## Results

### Allelic frequency and nucleotide analyses

DNAs from *H. pylori* strains from 44 Gambians (one strain/patient in 43 cases; three strains/patient in one case) yielded 42 unique MLST sequence types based on concatenated DNA sequences of seven housekeeping gene loci. Four pairs of strains yielded identical MLSTs. One pair was from consecutive unrelated patients whose biopsies were obtained on the same day. The other three pairs were also from unrelated patients who were biopsied between one week and two years apart. These exceptions aside, most alleles of individual genes occurred only once among the 46 strains, although identical alleles were found in 11 to 16 strains, depending on the gene. Except for the four pairs of strains that were identical at all loci (noted above), no pair of strains identical at one locus was identical at another of the seven loci tested (data not shown). The mean nucleotide diversity in the 7 genes was 2.9% (Table 2). The gene *trpC* had most strains with identical alleles (16 alleles); allele 1774 of *mutY* was most frequent (5 occurrences; 10.9%); the most diverse gene was *trpC* (mean nucleotide level diversity 4.6%); and the least diverse was *ureI* (1.2%, Table 2). No deletions or insertions were found in any of the analysed gene fragments.

**Table 2:** Genetic diversity of 46 Gambian isolates.

Locus	Dn	Ds	dn/ds	Diversity %
*atpA*	0.0006	0.1009	0.006	2.3
*efp*	0.0005	0.1059	0.0046	2.2
*mutY*	0.0098	0.1822	0.0536	4.5
*ppa*	0.0116	0.042	0.2761	2.1
*trpC*	0.0187	0.1513	0.1238	4.6
*ureI*	0.0052	0.0365	0.1428	1.2
*yphC*	0.0092	0.0924	0.0995	3.4
overall	0.0079	0.1016	0.1009	2.9

d_S_ and d_n_ are the average number of synonymous substitutions per synonymous site and non-synonymous substitutions per non-synonymous site, respectively.

### Analyses of selection

A nucleotide substitution in a coding region results either in a change or no change in the protein's amino acid sequence (non-synonymous (N), synonymous (S), respectively). The d_N_/d_S_ ratio in a population reflects genetic drift and selection operating on individual genes. All d_N_/d_S_ values in the seven housekeeping genes from Gambian *H. pylori* strains were close to zero (Table 2), indicating intense selection to maintain functions and amino acid sequences of the encoded proteins. This is expected for genes whose encoded proteins act within bacterial cells and provide important housekeeping functions.

### Phylogenetic analysis of Gambian strains, relative to those from elsewhere

**Figure 1:**
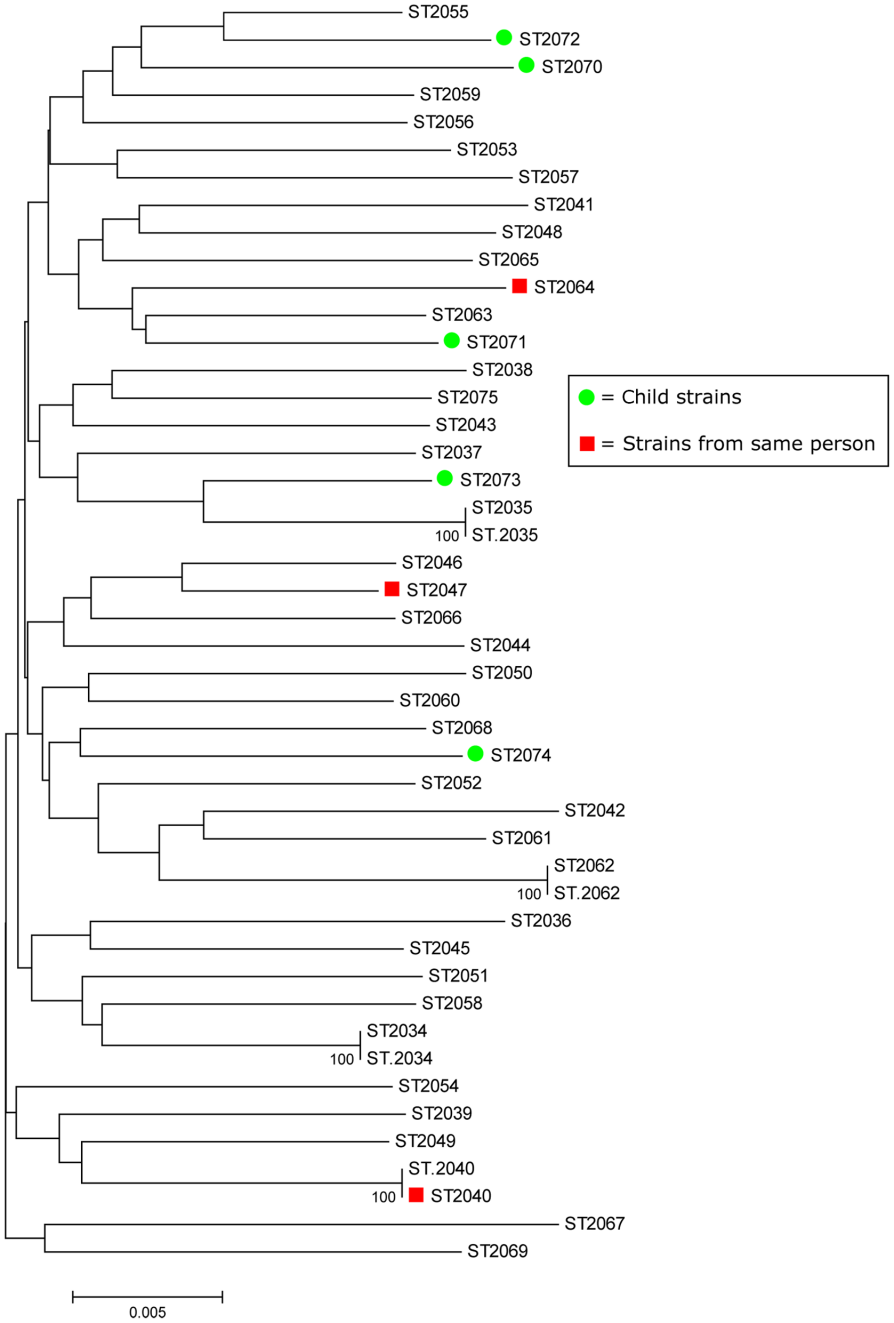
Evolutionary relationship among *H. pylori* strains isolated from The Gambia. Evolutionary history was inferred from concatenated sequences of the seven MLST housekeeping gene fragments (3406 bp) from 46 Gambian *H. pylori* using the neighbor-joining method. The analyses were conducted in MEGA5. The five strains from young children are identified with green circles. There was one subject with three different MLST types shown in red squares.

**Figure 2:**
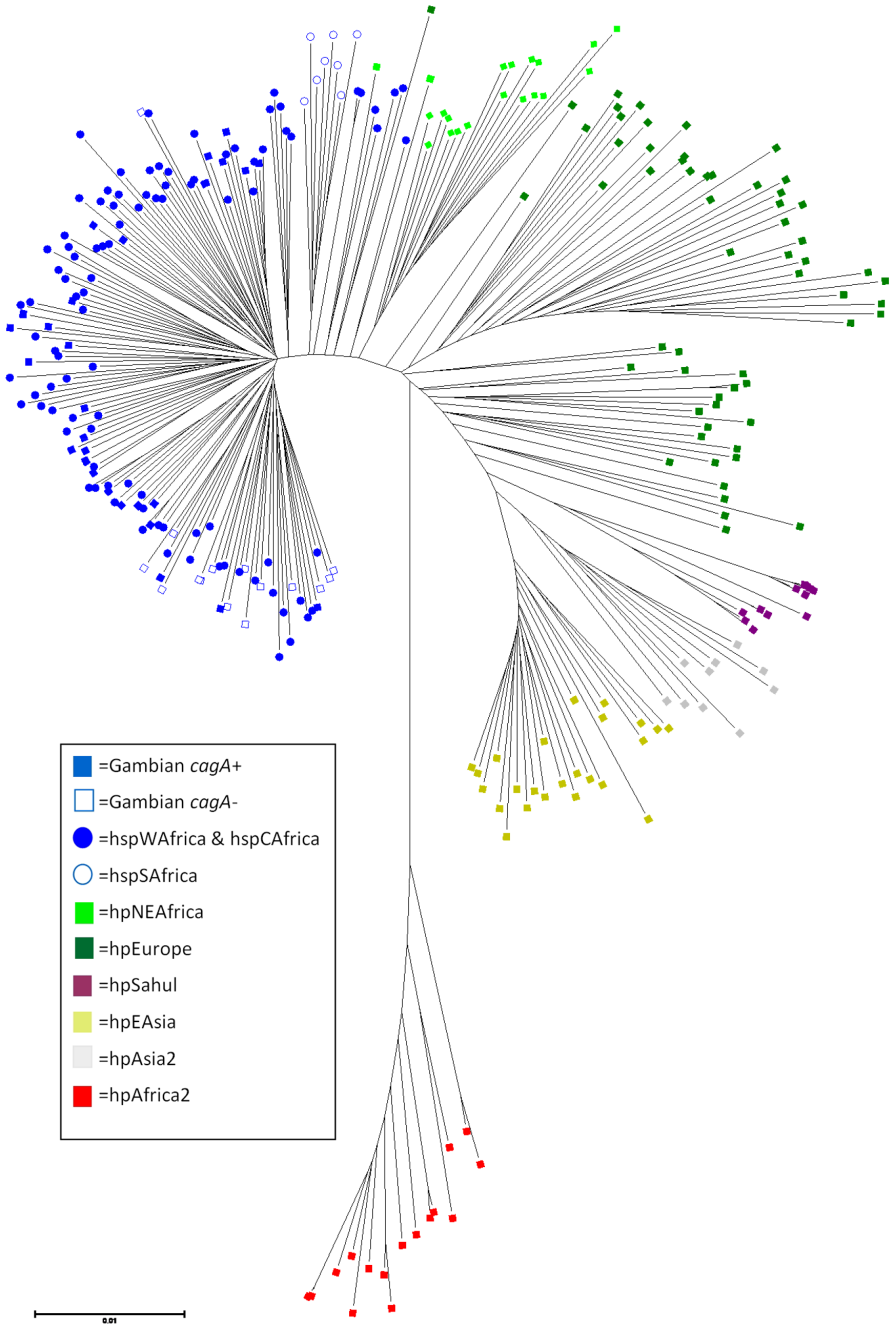
Evolutionary relationships among a global sample of *H. pylori* strains. The neighbor-joining tree was calculated from concatenated sequences of 246 globally representative *H. pylori* strains downloaded from MLST website (Http://pubmlst.org/Helicobacter) plus the 46 isolates studied here. Strains were colour-coded by population as follows: blue, hpAfrica1; light green, hpNEAfrica; dark green, hpEurope; grey, hpAsia2; purple, hpSahul; olive, hpEastAsia; red, hpAfrica2.

A phylogenetic tree reconstructed using concatenated sequences of the seven housekeeping genes (Figure 1) provided no evidence of association of particular clusters (clades) of strains with variables such as age of participant at time of endoscopy, endoscopic diagnosis, sex or district of residence within the Gambia. However, *cagA* ^+^ strains seemed to cluster separately from *cagA* ^-^ (Figure 2).

**Figure 3:**
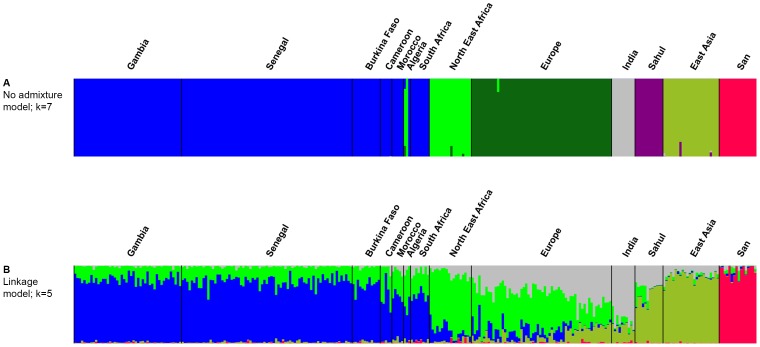
STRUCTURE analysis of 46 Gambian strains in relation to a representative sample of the global diversity in *H. pylori*. Gambian *H. pylori* were compared with strains from other previously assigned populations using no-admixture model (3A) and the linkage model (3B). Population are colour coded according to Figure 2. Each line represents an isolate and colours indicate its inferred modern (3A) or ancestral population(s) (3B).

Gambian strain sequences were also compared with sequences from 246 *H. pylori* strains selected from other informative human populations (African, European, Australian and Asian; Table 1) using neighbor-joining and Bayesian cluster analysis with both the no-admixture and linkage models of STRUCTURE. We found that all Gambian strains clustered within hpAfrica1 and were intermingled with strains from Senegal and Burkina Faso. hpAfrica1 strains formed a separate clade that was sister to strains from hpNEAfrica (Figure 2), with other European, Australian and Asian populations more distantly related. Under the no-admixture model, the number of populations (K) with the highest likelihood was seven, which corresponds to what is already known about the global structure of this bacterial species. Almost all Gambian isolates formed a homogeneous group together with other hpAfrica1 strains (Figure 3A). However, the linkage model showed that Gambian strains were more similar in their ancestral nucleotide composition to hspWAfrica strains from Senegal (Gambia's immediate neighbour) and Burkina Faso (population 3), whereas the North-East African ancestral component (ancestral Europe 2) was more apparent among those hpAfrica1 strains from more distant communities in Cameroon, Morocco, Algeria and South Africa (Figure 3B).

### Phenotypic heterogeneity of *H. pylori* in a single host

Bacterial colonies that differed markedly in morphology, seen among *H. pylori* cultured from biopsies from two patients with normal endoscopic gastroduodenal tract appearance (14 and 72 years of age), were used to test for *H. pylori* DNA level heterogeneity in individual hosts, perhaps equivalent to that seen previously in a European with a mixed *cagA* ^+^ and *cagA* ^-^ infection [31]. Seven colonies representative of the different morphologies were analysed from the 14 year old and 11 such colonies were analysed from the 72 year old. Three different MLST types were identified among the isolates from the first (14 year old) patient (MLST types 2040 in all four colonies from antrum, and 2047 and 2064 from one and two colonies, respectively, from the corpus; Figure 1). Each of these three MLST types was different from the two others in all seven gene loci tested. In the second (72 year old) patient, all 11 colonies (6 antrum, 5 corpus) were of the same MLST type. In neither case did the different colony morphologies that first encouraged analysis of multiple isolates from these two patients correspond to different MLST types.

## Discussion

Most detailed *H. pylori* population analyses to date have used strains from non-African countries, despite Africa's great importance for the emergence and evolution of humans and pathogens such as *H. pylori*. Here we sequenced the housekeeping genes of strains from The Gambia, one of the most detailed studies to date of a West African *H. pylori* population. These Gambian strains exhibited high nucleotide sequence diversity (mean, 2.9%), much as in isolates from other geographic regions [24], [32], with no obvious clustering of MLST types in particular age or disease groups. Our sequence data showed that Gambian *H. pylori* strains belong to the hpAfrica1 population and added greater resolution to the geographic distribution of this population in Africa. Comparison of ancestral nucleotides with those available from other African countries indicated some geographic differentiation, even within West Africa, as did a recent complementary analysis of many strains from Dakar, Senegal [30].

STRUCTURE analyses indicated that the contribution of ancestral nucleotides from other populations, mainly AE2 (which originated in North East Africa [22], to strains circulating in The Gambia was about 17%, which is the average of 3 independent linkage model runs at K = 5. These analyses also showed that Gambian strains are closely related to each other and belong to the hpAfrica1 population (Figures 3). The proportion of AE2 sequences in the Gambian strains is similar to, although marginally higher than, that found in strains from Senegal (19%) and Burkina Faso (23%), and much lower than in any other African country studied to date. This suggests a history of limited admixture (recombination) with strains from elsewhere, as also noted by others [30].

In the case of Gambian *H. pylori*, recombination from other non-hpAfrica1 strains could possibly have occurred after contact with Europeans or North Africans. However, the population (hpEurope) carried by Europeans is itself a hybrid (recombinant), consisting of roughly equal contributions from AE1 and AE2, originating in Central Asia and North-East Africa, respectively [30], [33]. Since no trace of AE1 was found in our sample, we conclude that the source of the AE2 nucleotides in the Gambia is unlikely to have resulted from hpEurope strains following European colonization of African lands during the last six centuries. This also sheds light on the nature of the centuries-long contact between Gambians and Europeans/North Africans. In South Africa, hpEurope was found in the stomachs of indigenous Africans from several ethnicities [33], pointing to a more extensive association between colonizing Europeans and local populations than in the Gambia, where Europeans never made up a significant part of the total population.

Therefore, we propose that the Gambian *H. pylori*'s AE2 nucleotides were derived from contact with Nilo-Saharan speakers, perhaps when these people migrated westwards from the Nile valley across the Sahara during the Holocene humid period, 6,000–9,000 or more years ago [25]. In accord with this, hpNEAfrica sequences have also been detected in strains from Cameroon [25], northern (predominantly Muslim) Nigeria [22] and Algeria [22] (Figure 3B). Alternatively, since hpAfrica1 and hpNEAfrica are sister phylogenetic groups (Figure 2), the observed AE2 ancestry among Gambian, Senegalese and Burkina Faso strains could also be attributed to background linkage disequilibrium, where ancestral nucleotides were already present in the common ancestor of both populations.

### Identical MLST types

We found four pairs of isolates with identical MLST types. Three of these pairs were from people whose gastric biopsies were obtained between one week and two years apart and processed in the laboratory on different dates. They thus are likely to reflect occasional carriage of closely related strains by unrelated members of a community. We also suggest this explanation for the one matched pair from patients who had gastric biopsies obtained consecutively on the same day, since our endoscopes were rigorously cleaned and washed after each use (see Materials and Methods).

Although none of the strain pairs with identical MLSTs were from persons from the same village or with the same family names, further study will be needed to learn if these people had ever lived in the same extended family compound, village or district, or had some other connection, vs. if such identical MLST types reflect some other factor such as The Gambia's small size and easy hospitality to strangers, and/or frequent community transmission of *H. pylori* in developing country settings [17].

Given the lack of obvious connection between these paired strains, genome-wide analyses of their patterns of micro-sequence divergence vs. conservation could also be highly informative, especially in genes for secreted proteins involved in host interaction and more subject to diversifying selection.

### Heterogeneity of *H pylori* strains within one stomach

Among isolates from the two patients tested for possible *H. pylori* heterogeneity, all colonies from one patient were identical by MLST whilst the other patient had three distinct MLST types, consistent with other findings [15], [16], [31]. The sequence types of these three strains were different in all seven of the gene loci scored, thereby suggesting co-infection by unrelated strains [27], [34].

Our study indicated that Gambian *H. pylori* are not particularly clonal, in accord with patterns seen in other non-African populations. Since the MLST types of strains from young children were intermingled with those from adults, there may also not be any special strain type uniquely able to initiate infection in naive infant stomachs. We also note that our Gambian strains showed more genetic similarity with strains from Senegal and Burkina Faso (both countries in far Western Africa) than from elsewhere, reflecting again geographic partitioning of *H. pylori*.
